# DNA Polyelectrolyte Multilayer Coatings Are Antifouling and Promote Mammalian Cell Adhesion

**DOI:** 10.3390/ma14164596

**Published:** 2021-08-16

**Authors:** Omar Abdelaziz Ouni, Guruprakash Subbiahdoss, Andrea Scheberl, Erik Reimhult

**Affiliations:** Department of Nanobiotechnology, Institute of Biologically Inspired Materials, University of Natural Resources and Life Sciences (BOKU), 1190 Vienna, Austria; omarabdellazizouni@gmail.com (O.A.O.); andrea.scheberl@boku.ac.at (A.S.); erik.reimhult@boku.ac.at (E.R.)

**Keywords:** implants, DNA, chitosan, antifouling, osteoblasts, *Staphylococcus*, titanium, PMMA

## Abstract

The ability of bacteria to adhere to and form biofilms on implant surfaces is the primary cause of implant failure. Implant-associated infections are difficult to treat, as the biofilm mode of growth protects microorganisms from the host’s immune response and antibiotics. Therefore, modifications of implant surfaces that can prevent or delay bacterial adhesion and biofilm formation are highly desired. In addition, the attachment and spreading of bone cells are required for successful tissue integration in orthopedic and dental applications. We propose that polyanionic DNA with a negatively charged phosphate backbone could provide a dual function to repel bacterial adhesion and support host tissue cell attachment. To this end, we developed polyelectrolyte multilayer coatings using chitosan (CS) and DNA on biomaterial surfaces via a layer-by-layer technique. The assembly of these coatings was characterized. Further, we evaluated staphylococcal adhesion and biofilm growth on the coatings as well as cytotoxicity for osteoblast-like cells (SaOS-2 cells), and we correlated these to the layer structure. The CS-DNA multilayer coatings impaired the biofilm formation of *Staphylococcus* by ~90% on both PMMA and titanium surfaces. The presence of cationic CS as the top layer did not hinder the bacteria-repelling property of the DNA in the coating. The CS-DNA multilayer coatings demonstrated no cytotoxic effect on SaOS-2 cells. Thus, DNA polyelectrolyte multilayer coatings could reduce infection risk while promoting host tissue cell attachment on medical implants.

## 1. Introduction

Implant surgeries have grown in the past decades. Studies show a dramatic increase in the need for hip and knee primary or revision surgeries [1]. However, the use of implants is restricted by complications due to implant-associated infections (IAI). Almost 20% of implant failures are caused by IAI [2]. IAI are difficult to treat, as microbes, especially bacteria, attach to implants and form biofilms. In biofilms, the extracellular polymeric matrix protects the bacteria from the host’s immune system and antibiotics [3]. Biofilm formation involves different steps: the formation of the conditioning layer, the adherence of the bacteria, the secretion of extracellular polymeric substances, and three-dimensional matrix development followed by maturation and dispersion [4]. A broad range of bacteria can cause IAI. However, *Staphylococcus epidermidis* and *Staphylococcus aureus* account for almost 70% of infections [4]. In addition, aseptic loosening represents 18% of implant failures, which is caused by gaps at the prosthesis–bone interface, poor bone in-growth, or bone deposition on implant surfaces [2,5]. It is essential for orthopedic and dental implants to establish a robust implant–bone interface. Bone cells must adhere, spread, proliferate, and differentiate for successful tissue integration [6,7]. 

Bacterial adhesion and biofilm formation can occur on almost all clinically used implant materials. Common implant materials include metals (titanium, stainless steel, etc.) and polymers such as PMMA, polyethylene, and polytetrafluorethylene [8], among which titanium is considered the gold standard implant material, widely used in dental and orthopedic implants, heart valves, and vascular stents [9,10,11]. However, bacterial adhesion and biofilm formation occur on Ti surfaces [7]. The physicochemical properties, i.e., chemical composition, surface roughness, surface energy, and surface charge of implant surfaces, determine microbial adhesion [12]. Thus, the modification of implant surfaces could prevent bacterial adhesion and biofilm formation. 

DNA is a negatively charged polymer with phosphate groups on the backbone, which has been shown to prevent fouling [13,14]. We recently demonstrated that DNA coatings applied to stainless steel via the layer-by-layer (LbL) technique reduced inorganic and microbial fouling from tap water when incubated statically or in flow. DNA coatings impaired biofilm formation by 93% on stainless steel from tap water and reduced the initial attachment of *Staphylococcus* and *Pseudomonas* on glass surfaces [13]. 

DNA is a biopolymer, which is stable and improves the biocompatibility of biomaterials when coated via the LbL technique [15,16,17]. In this study, we propose that DNA coatings can prevent bacterial adhesion and biofilm formation while promoting mammalian cell attachment and spreading. We used an alternating multilayer of DNA and chitosan (CS) coating formed using LbL deposition on PMMA and titanium surfaces characterized by QCM-D and contact angle measurements to verify our hypothesis. CS is a cationic, linear polysaccharide derived from chitin that possesses antimicrobial properties [18,19]. The substrates were primed for the LbL deposition of CS and DNA by the formation of an initial multilayer using highly charged polyelectrolytes poly(ethylene imine) (PEI) and poly(styrene sulfonate) (PSS) to achieve a stable coating. Bacterial adhesion and biofilm growth of *S. epidermidis* and *S. aureus* were studied on the CS-DNA multilayer coatings using fluorescence microscopy. Further, we investigated the cytotoxicity by growing osteoblast-like cells on CS-DNA multilayer-coated surfaces. 

## 2. Materials and Methods

### 2.1. Biomaterial Surfaces

Poly(methyl methacrylate) (PMMA) and titanium (Ti) plates purchased from Goodfellow Cambridge, UK, with the size of 10 mm × 10 mm, were used for the experiments. Surfaces were cleaned with sonication using ethanol for 10 min, were washed thoroughly with Milli-Q water, and were dried using nitrogen gas. Pre-cleaned plates were stored in sterile containers until further use.

### 2.2. Build-Up of CS-DNA Multilayer Coatings 

The pre-cleaned PMMA and Ti plates were first immersed in poly(ethyleneimine) (PEI, 181978 Sigma Aldrich, Vienna, Austria) solution at a concentration of 1 mg/mL in 10 mM phosphate-buffered saline (PBS) for 5 min, were washed with Milli-Q water by rinsing for 3 s, were then immersed in poly(styrene sulfonate) (PSS, 243051 Sigma Aldrich) solution at a concentration of 1 mg/mL in PBS for 5 min, and were washed again with Milli-Q water. This immersion cycle was repeated until 3 bilayers (PEI-PSS)_3_ were obtained. These plates were then dipped in a chitosan (CS, 419419 Sigma Aldrich) solution consisting of 0.6% *w*/*v* dissolved in a 1% (*v*/*v*) acetic acid (45731, Fluka Analytical, Munich, Germany) solution for 5 min, were rinsed with Milli-Q water for 3 s, and were then dipped in DNA (DNA sodium salt from salmon testes, D1626 Sigma Aldrich) solution at a concentration of 1 mg/mL in PBS for 5 min. This immersion cycle was repeated, and the build-up of CS-DNA multilayer coatings continued until 4 bi-layers (CS-DNA)_4_ were obtained. All of the polymer solutions were prepared and coated on surfaces one day prior to the experiments and were stored at room temperature. The multilayer-coated plates were denoted as Ti/PMMA-LbL(CS-DNA)_4_ (DNA as top layer) and Ti/PMMA-LbL(CS-DNA)_4_-CS (CS as top layer). 

### 2.3. Characterization of the Polyelectrolyte Multilayer Formation 

Quartz crystal microbalance with dissipation (QCM-D, Q-Sense AB, Gothenburg, Sweden) monitoring on a Q-Sense E4 system was used to monitor the LbL self-assembly process; the changes in resonance frequency and the dissipation of an oscillating quartz crystal were used to quantify changes in the hydrated mass and viscoelastic properties of the coating. In brief, the titanium-coated sensor crystals (QSX 310 Titanium) were stored in 1% Hellmanex II (Sigma Aldrich) for 30 min, washed with Milli-Q water, and dried using nitrogen gas. Subsequently, the sensors were sonicated in 99% ethanol for 10 min, rinsed with Milli-Q water, dried with nitrogen gas, and subjected to UV-ozone cleaning for 10 min. The assembly of the multilayer on the sensor was conducted by injecting polymer solutions into the flow cell in the same sequence described in the previous section to build up CS-DNA multilayers on Ti/PMMA via dip-coating. Briefly, PEI and PSS solutions were injected alternately three times each at a flow rate of 0.05 mL/min. The CS and DNA solutions were then injected alternately four times each at the same flow rate for 5 min. Milli-Q water was injected for 5 min at 0.05 mL/min between each adsorption to remove the loosely bound molecules. All measurements took place at 25 °C. Each sensor crystal was observed using the Q-Soft software at different overtones (3rd, 5th, 7th, 9th, and 11th). The adsorbed mass change Δm during the assembly of the film was calculated using the Sauerbrey equation [20,21]:

Δm=−C×Δf/n

where C is the constant of value 17.7 ng cm^−2^ Hz^−1^, Δf is the frequency change, and n is the overtone.

Coating homogeneity was assessed using contact angle measurements and scanning electron microscopy (SEM).

The wettability of the surfaces was determined by water contact angle measurements using a Krüss DSA 25 (Krüss, Hamburg, Germany) at room temperature. Surfaces were cleaned as described above. Measurements were performed with Milli-Q water. Contact angles were calculated from the images using Krüss software. Each value was obtained by averaging three droplets on one surface and with a minimum of three replicate surfaces.

Scanning electron microscopy was performed on the multilayer-coated plates to examine the presence of coatings. Imaging was performed in secondary electron mode at high vacuum using an Apreo VS SEM (Thermo Scientific, city, Eindhoven, The Netherlands) at 5 kV after scratching the coating to produce a contrast between coated and uncoated areas. 

### 2.4. Bacterial Growth Conditions and Harvesting

The *Staphylococcus aureus* ATCC 12598 (DSM 20372) and *Staphylococcus epidermidis* ATCC 35984 (DSM 28319) that were used in this study were obtained from the DSMZ-German collection of microorganisms and cell culture GmbH. Overnight cultures were prepared by transferring a single colony from an agar plate to 10 mL of tryptone soy broth (TSB) in a 50 mL Erlenmeyer flask. The suspension was incubated overnight at 37 °C, with shaking at 100 RPM. The bacterial cells were harvested through centrifugation at 5000 RPM for 5 min and were adjusted to a dilution of 0.5 OD at 600 nm in fresh TSB. 

### 2.5. Bacterial Adhesion and Biofilm Growth Assay

CS-DNA multilayer-coated PMMA and Ti plates were placed in sterile tissue culture polystyrene (TCPS, Agilent Technologies, Vienna, Austria) well plates. Each well was filled with 4 mL bacterial suspension and was cultured at 37 °C for 24 h. At 2 h and 24 h, the bacterial suspension was removed, and the wells were washed with PBS to remove unbound bacteria. Subsequently, vitality staining solution (3.34 mM SYTO 9 and 20 mM propidium iodide, Invitrogen, Thermo Fischer Scientific, Austria) in PBS was added to the wells and was incubated for 15 min in the dark at room temperature. The surfaces were then rinsed with PBS to remove the unbound staining, followed by observation using fluorescence microscopy (Nikon Eclipse TE2000, Nikon Europe B.V., Vienna, Austria) to characterize the viability of the adherent bacteria on the coated plates. Live bacteria with intact cell membranes emit green, whereas dead bacteria with damaged membranes emit red. The uncoated PMMA and Ti plates were used as control.

### 2.6. Mammalian Cell Adhesion

SaOS-2 Osteosarcoma cells ACC 243 (DSMZ-German Collection of Microorganisms and Cell Culture GmbH, Braunschweig, Germany) were routinely cultured in Dulbecco’s modified Eagle’s Medium (DMEM) with low glucose supplemented with 10% fetal calf serum (FCS) and 20 mM HEPES. SaOS-2 cells were maintained in a T75 cell culture flask at 37 °C in a humidified 5% CO_2_ atmosphere and were harvested at 95% confluency using TrypLE. The harvested cells were stained with a Trypan blue solution, were counted using a Countess^®^ automated cell counter (Invitrogen, Thermo Fischer Scientific, Austria), and were subsequently diluted to a concentration of 5 × 10^4^ cells/mL.

To determine SaOS-2 cell growth on multilayer coatings, 2 mL of SaOS-2 cell suspension with a concentration of 5 × 10^4^ cells/mL was seeded to each TCPS well containing coated and uncoated PMMA plates. SaOS-2 cells were incubated at 37 °C in a humidified 5% CO_2_ atmosphere for 48 h. Subsequently, the cells were fixed with Roti Histofix for 10 min and were rinsed with PBS. Cells were treated with 0.5% Triton X-100 in PBS (1 mL per well) for 3 min. After rinsing three times with PBS, the cells were stained for 10 min with 1% DAPI (Sigma Aldrich) and 0.2% TRITC-phalloidin (Sigma Aldrich) in PBS, and they were rinsed with PBS and observed using fluorescence microscopy. The surface coverage by SaOS-2 cells was calculated using the ImageJ software (U.S. National Institutes of Health, Bethesda, MD, USA).

## 3. Results

### 3.1. S-DNA Dip-Rinse Cycles Formed Linearly Growing Polyelectrolyte Multilayers

**Figure 1:**
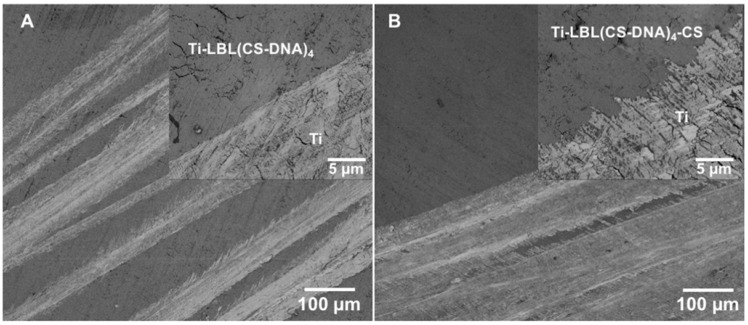
Scanning electron micrographs of multilayer coatings on Ti surfaces. The coating was removed by performing a scratch using a tweezer. (**A**) Representative micrograph comparing Ti-LbL(CS-DNA)_4_ coating (dark region) and the uncoated Ti surface (light region). (**B**) Representative micrograph comparing Ti-LbL(CS-DNA)_4_-CS coating (dark region) and the uncoated Ti surface (light region). The insets are high magnification images. The presence of a coating can be clearly differentiated from the uncoated Ti surface at the border where the coating was removed.

In the present work, multilayer coatings on PMMA or Ti surfaces were fabricated by the alternate adsorption of polycations (PEI or CS) and polyanions (PSS or DNA) through electrostatic interaction via the LbL technique. The initial three bilayers were formed by successive immersion and rinse cycles of PEI and PSS, which were followed by another four bilayers formed using CS and DNA. Figure 1 shows SEM images of the multilayer coatings taken in secondary electron mode under high vacuum at 1 kV. The presence of a coating by immersion cycles of PEI and PSS until 3 bilayers (PEI-PSS)_3_ followed by immersion cycles of CS and DNA until 4 bi-layers (CS-DNA)_4_ were obtained can be clearly differentiated from the uncoated Ti surface where the coating was removed after deposition (Figure 1).

**Figure 2:**
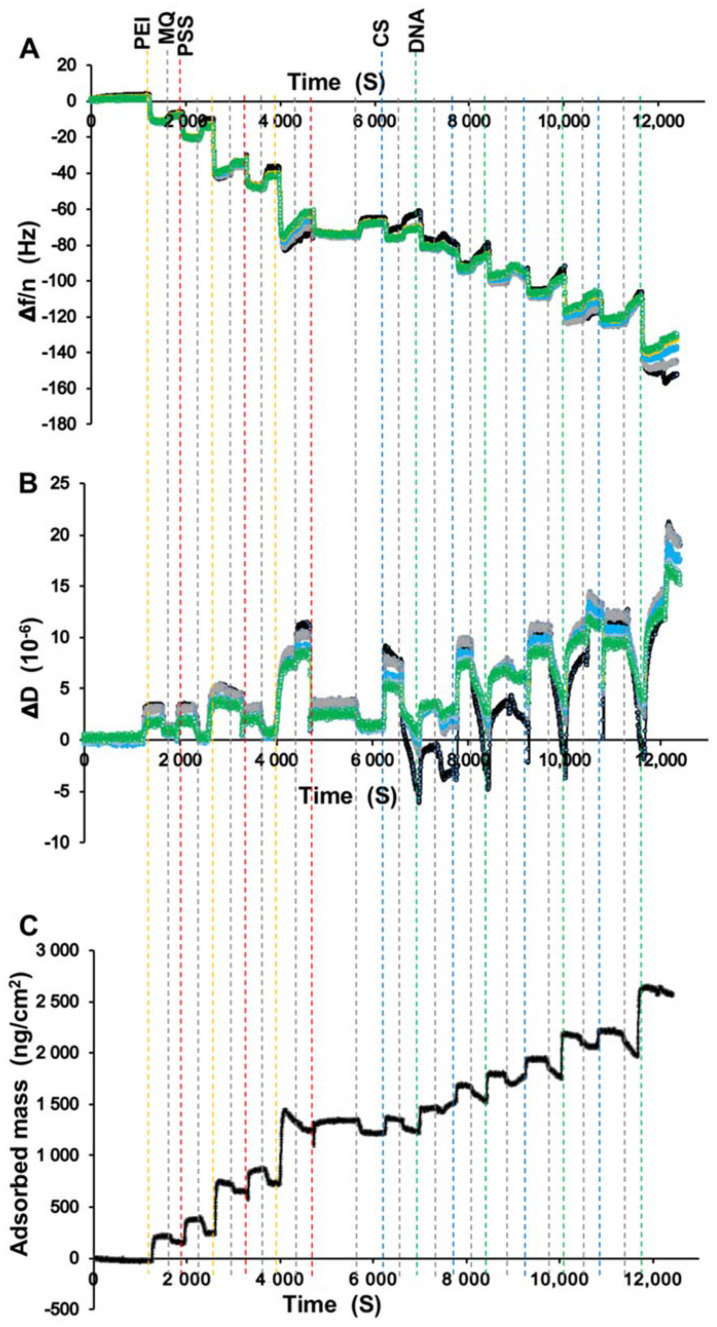
QCM-D data showing the evolution of (**A**) the frequency shift, Δf/n, and (**B**) the dissipation ΔD during the film build-up for the overtones (n= 3, 5, 7, 9, and 11). The Δf/n decrease observed after each successive layer of polyelectrolytes PEI (yellow vertical dashed lines), PSS (red), CS (blue), and DNA (green) shows a representative example of the film build-up. The Δf/n increase observed after the injection of Milli-Q water (grey vertical dashed lines) indicates the removal of weakly adsorbed excess polymer. The ΔD increased rapidly upon the addition of each layer but decreased much more strongly than the Δf/n upon rinsing, indicating the removal of primarily loosely coupled surface polymer. (**C**) The adsorbed mass of the film calculated from Δf/n according to the Sauerbrey equation as a function of time.

The build-up of LbL coatings deposited on the titanium-coated sensor crystal surface was followed by monitoring the changes in the resonant frequency (Δf) and the energy dissipation (ΔD) at the overtones (n) using QCM-D (Figure 2). Figure 2A shows the evolution of the overtone-normalized frequency shift Δf/n during the film build-up for different overtones (n= 3, 5, 7, 9, and 11). The Δf/n decrease observed after each successive layer of electrolytes demonstrates a gradual growth of the polymeric film. During Milli-Q water washing, loosely bound molecules were removed, causing an increase in Δf/n (Figure 2A). The dissipation shifts grew rapidly in tandem with the decrease in Δf/n (Figure 2B). The removal of loosely bound molecules during the rinsing steps resulted in decreased dissipation, which was much stronger than the corresponding increase in Δf/n (Figure 2B). The significant frequency decrease but low remaining dissipation increase of the rinsed films indicates that very loosely coupled polymers at the top of the LbL were removed during rinsing, leaving a relatively rigid and dense film.

A superposition of the frequency shifts at different overtones suggests that all films are quite rigid (Figure 2A). The responses of Δf/n and ΔD are small, and their changes in a linear fashion with the addition of layers indicate thin, rigid films deposited on the titanium-coated sensor crystals. A measure of $\Delta D/\left(-\Delta f/n\right)<0.4\times 10^{-6}$ Hz^−1^ is often invoked to distinguish between rigid films that are modeled well by the Sauerbrey equation and viscoelastic films that require viscoelastic data modeling to determine the adsorbed mass [22,23]. In all of our experiments, we observed a ratio of $\Delta D/\left(-\Delta f/n\right)<0.2\times 10^{-6}$ Hz^−1^, indicating a rigid layer. Hence, we used the Sauerbrey equation to calculate the mass of the hydrated polymer film after each adsorption step (Figure 2C) [21]. A total mass of 2560 ± 380 ng/cm^2^ was estimated for the complete 14-layer LbL(CS-DNA)_4_ polyelectrolyte multilayer (Figure 2C). 

### 3.2. DNA-Terminated Multilayers Are Hydrophilic Compared to Uncoated Surfaces and Cationic Polymer-Terminated Multilayers

**Figure 3:**
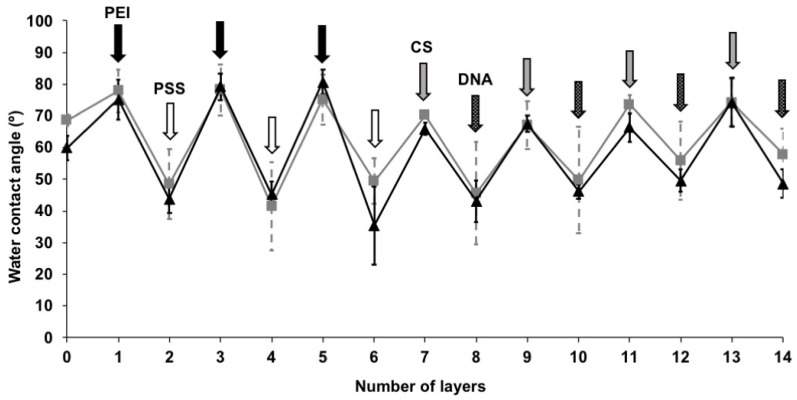
Water contact angles of (PEI-PSS)_3_ followed by (CS-DNA)_4_ multilayer films on PMMA (grey line) and Ti (black line) surfaces. The error bars represent the standard deviation over three replicate surfaces and three measurements per surface.

After each adsorption step, the water contact angle was measured and showed how the hydration changed with each new deposition (Figure 3). The uncoated Ti and PMMA surfaces showed intermediate hydrophilicity after sonication in EtOH. Ti had a contact angle of 60° ± 4° and a PMMA of 69° ± 2°, respectively. As expected, a CS terminated multilayer presented a surface with a similar contact angle, which was more hydrophobic. Both the DNA-terminated multilayers showed lower contact angles, but a higher contact angle was observed on the PMMA than on the Ti substrates, even after 14 layer depositions (Ti-LbL(CS-DNA)_4_ 48.6° ± 4.4°, PMMA-LbL(CS-DNA)_4_ 57.9° ± 8.3°). These results demonstrate that the last adsorbed polymer layer dominates the top layer surface properties, as expected for a linearly growing LbL film.

### 3.3. CS-DNA Multilayer Coatings Suppressed Biofilm Formation

**Figure 4:**
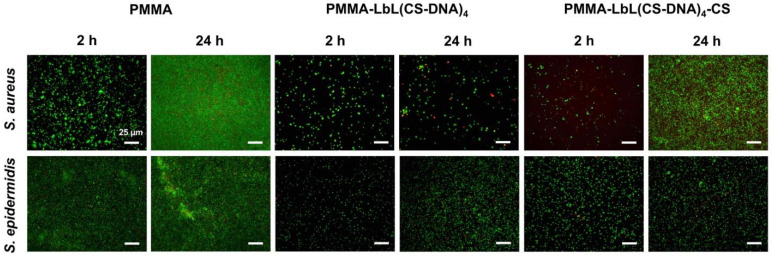
Representative fluorescence microscope images of adherent *S. aureus* ATCC 12598 and S. *epidermidis* ATCC 35984 on PMMA, PMMA-LbL(CS-DNA)_4_, and PMMA-LbL(CS-DNA)_4_-CS surfaces after incubation at 37 °C for 2 h and 24 h, respectively. Bacteria were stained using vitality staining solution (3.34 mM SYTO 9 and 20 mM propidium iodide in PBS) and were incubated for 15 min in the dark at room temperature. The scale bar denotes 25 µm.

To investigate the effect of the CS-DNA multilayer coatings on bacterial adhesion and biofilm formation, coated and uncoated PMMA or Ti surfaces were incubated with suspensions of staphylococci (*S. aureus* ATCC 12598 and *S. epidermidis* ATCC 35984) at 37 °C for 24 h. Bacteria attached to the surfaces were imaged by fluorescence microscopy at 2 h and 24 h after live–dead staining. Representative fluorescence images of adherent *S. aureus* and *S. epidermidis* on coated and uncoated PMMA surfaces are shown in Figure 4. Polyelectrolyte multilayer films with a DNA top layer (PMMA-LbL(CS-DNA)_4_) and with a CS top layer (PMMA-LbL(CS-DNA)_4_-CS) are compared.

**Figure 5:**
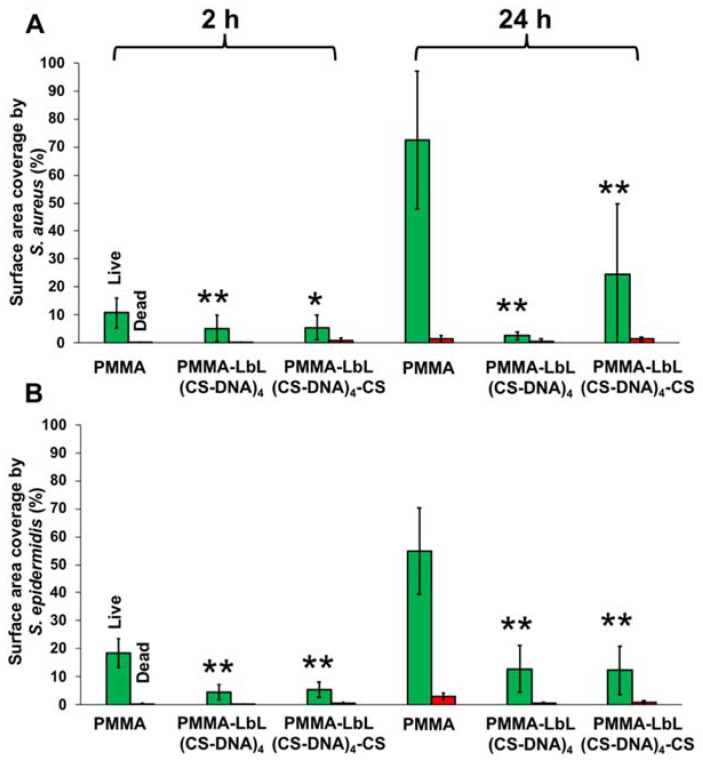
Surface coverage of adherent live (green bars) and dead (red bars) bacteria. (**A**) *S. aureus* ATCC 12598 and (**B**) *S. epidermidis* ATCC 35984 on uncoated and multilayer-coated PMMA surfaces after incubation at 37 °C for 2 h and 24 h. PMMA-LbL(CS-DNA)_4_ has a DNA top layer, and PMMA-LbL(CS-DNA)_4_-CS has a CS top layer. The error bars represent the standard deviation over three biological replicates. ANOVA tests were performed, followed by a Tukey’s HSD post hoc test, and a *p*-value < 0.05 was considered significant. ** denotes significance (*p* < 0.01) compared to uncoated PMMA, and * denotes significance (*p* < 0.05) compared to uncoated PMMA.

The initial adhesion and biofilm formation of the staphylococci were quantified by analyzing the surface coverage from the fluorescence images using ImageJ. Both polyelectrolyte multilayer coatings reduced the bacterial adhesion (2 h) and the biofilm growth (24 h) of both *S. aureus* and *S. epidermidis* significantly (*p* < 0.01) compared to the uncoated PMMA surfaces (Figure 5). At 24 h, multilayer coatings with a DNA top layer reduced the *S. aureus* surface coverage from 72% (bare PMMA) to 2.6% and the *S. epidermidis* surface coverage from 55% on bare PMMA to 12%. When CS was used as the top layer, the bacterial attachment and the biofilm growth were still significantly (*p* < 0.05) reduced on the PMMA surfaces (Figure 5), indicating that the antifouling properties of DNA remain even when nominally covered by a cationic CS layer. When the bacteria attached to the surface was imaged by fluorescence microscopy at 2 h and 24 h after live–dead staining, the presence of CS did not significantly increase bacterial killing on PMMA surfaces. 

**Figure 6:**
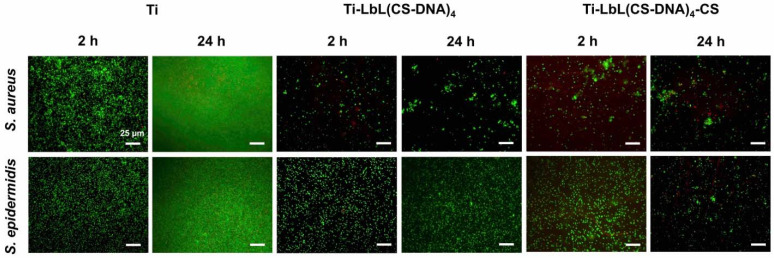
Representative fluorescence microscope images of adherent *S. aureus* ATCC 12598 and *S. epidermidis* ATCC 35984 on Ti, Ti-LbL(CS-DNA)_4_, and Ti-LbL(CS-DNA)_4_-CS surfaces after incubation at 37 °C for 2 h and 24 h, respectively. Bacteria were stained using vitality staining solution (3.34 mM SYTO 9 and 20 mM propidium iodide in PBS) and were incubated for 15 min in the dark at room temperature. The scale bar denotes 25 µm.

**Figure 7:**
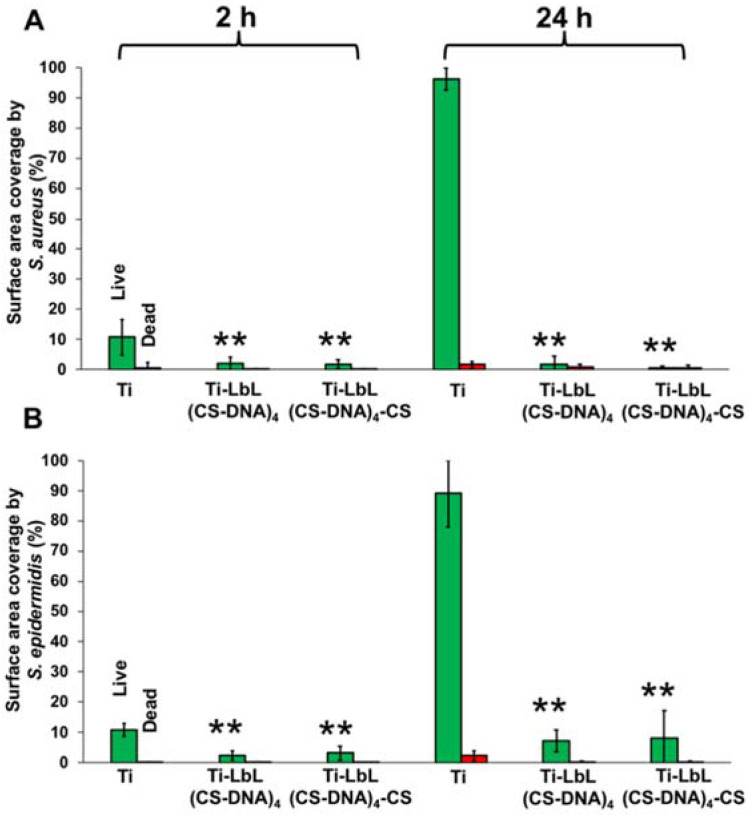
Surface coverage of adherent live (green bars) and dead (red bars) bacteria. (**A**) *S. aureus* ATCC 12598 and (**B**) *S. epidermidis* ATCC 35984 on uncoated and multilayer-coated Ti surfaces after incubation at 37 °C for 2 h and 24 h. Ti-LbL(CS-DNA)_4_ has a DNA top layer, and Ti-LbL(CS-DNA)_4_-CS has a CS top layer. The error bars represent the standard deviation over three biological replicates. ANOVA tests were performed followed by a Tukey’s HSD post hoc test, and a *p*-value < 0.05 was considered significant. ** denotes significance (*p* < 0.01) compared to uncoated Ti.

Fluorescence images of adherent *S. aureus* and *S. epidermidis* at 2 h and 24 h on the coated and uncoated Ti surfaces are shown in Figure 6. Similar to the results on PMMA surfaces, all of the polyelectrolyte multilayer coatings significantly (*p* < 0.01) reduced the surface coverage of bacteria on the Ti surfaces. At 24 h, the multilayer coatings with DNA as the top layer reduced the surface coverage of *S. aureus* on Ti from 95% (bare Ti) to 2% and of *S. epidermidis* on Ti from 90% (bare Ti) to 6% (Figure 7). CS as the top layer did not show a significant effect on the antimicrobial activity on the Ti surfaces.

### 3.4. CS-DNA Multilayer Coatings Supported Osteoblast-like Cell Attachment and Spreading

**Figure 8:**
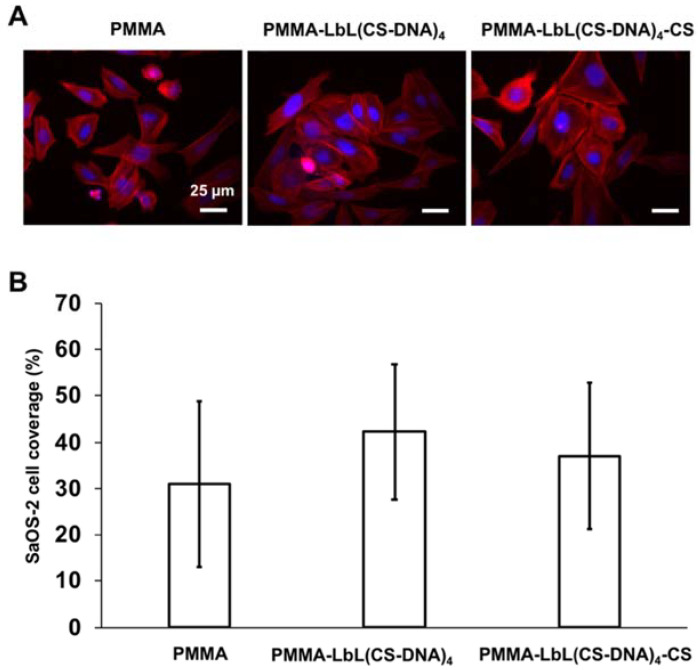
(**A**) Fluorescence microscopic images of SaOS-2 cells after 48 h growth on PMMA, PMMA-LbL(CS-DNA)_4,_ and PMMA-LbL(CS-DNA)_4_-CS surfaces. SaOS-2 cells were stained with PBS containing DAPI and TRITC-phalloidin. The scale bar denotes 25 µm. (**B**) Surface coverage by SaOS-2 cells after 48 h of growth on uncoated and multilayer-coated PMMA surfaces. The error bars represent the standard deviation over three replicate samples and six images per sample with separately cultured SaOS-2 cells. ANOVA tests were performed, and no significant differences were observed between surfaces.

The response of SaOS-2 cells after 48 h of growth on uncoated and multilayer-coated PMMA surfaces is shown in Figure 8. The spreading of the cells showed high variability on all surfaces, but the CS-DNA multilayer coatings did not seem to affect the SaOS-2 cell attachment and the spreading on the PMMA surfaces compared to the bare surface control (Figure 8A). Similar SaOS-2 cell morphology was observed on all of the surfaces (Figure 8A, more images in Figure S1). No significant difference in SaOS-2 cell surface coverage was observed between uncoated and multilayer-coated PMMA surfaces (Figure 8B). The presence of CS as the top layer in PMMA-LbL(CS-DNA)_4_-CS did not negatively affect the attachment and spreading of SaOS-2 cells.

## 4. Discussion

In this study, titanium and PMMA were used as substrates because of their relevance as materials for dental and orthopaedic implants. These biomaterials support host tissue cell attachment and spreading, but they also allow bacterial adhesion and biofilm growth. The adhesion of bacteria to a biomaterial surface depends on the physicochemical properties, i.e., roughness, chemical composition, charge, and hydrophobicity of the surface [24,25]. Therefore, in this study, we modified the implant surfaces (PMMA and Ti) with DNA coatings via the LbL technique. 

The physicochemical properties of the underlying substrate affect the structure, stability, and properties of LbL films. They play a crucial role in the deposition of the initial layers, affecting both the coating homogeneity and final stability [26,27]. Hence, priming a substrate with polyelectrolytes that promote homogeneity and stability before building a functional LbL film is recommended.

In our previous study, we used PEI as an anchoring layer followed by the sequential adsorption of DNA and PEI for up to six bilayers on stainless steel surfaces, resulting in the formation of a soft film [13]. In this study, to achieve more stable multilayer coatings, the highly positively charged polycation PEI was used as the first anchoring layer followed by the highly negatively charged polyanion PSS. A total of three bilayers of PEI-PSS were used to prime the surface. This was followed by the adsorption of four CS and DNA bilayers on the titanium surfaces. The adsorbed polyelectrolyte layers were thin, robust, and relatively rigid (Figure 2), with the DNA-terminated coatings being marginally more hydrophilic than the uncoated surfaces (Figure 3). We hypothesized that the difference in the film properties between our earlier and current work is mainly attributed to the different substrates and initial bilayers. To test the hypothesis, we coated a PEI layer as the anchoring layer followed by the sequential adsorption of DNA and PEI for up to six bilayers on titanium-coated sensor crystals surface using QCM-D. The build-up of the LbL coatings deposited on the titanium surface was followed by monitoring the changes in the frequency (Δf) and dissipation (ΔD) at different overtones (n) (Figure S2). Frequency shifts at different overtones are superimposed at the beginning of the LbL assembly, suggesting the formation of rigid films up to three bilayers. Later, as the film grows, divergence in the frequency shifts at different overtones become more prominent. Accordingly, the dissipation increased significantly for 4–6 bilayers compared to its low value for the initial three bilayers. These data suggest that the PEI-DNA polyelectrolyte films become structurally weaker, more hydrated, and have a higher viscous modulus after the first three bilayers. In contrast, when highly charged polyelectrolytes (PEI-PSS bilayers) were used as the initial three bilayers, more compact, rigid films were formed. The total amount of adsorbed mass was ~8 times lower than the PEI-DNA polyelectrolyte films deposited on the titanium-coated sensor crystals. We suggest that the mass difference can mainly be attributed to the higher water content and lower structural integrity of the former.

In a different study, Trybala et al. [27] investigated the effect of surfaces of various materials (titanium, stainless steel 316L, and silicon plates) and surface roughness on the adsorption of polyelectrolytes. In addition, they evaluated the effect of PEI as the anchoring layer to the substrate surface on the formation of polyelectrolyte multilayer films. The amount of polymer that was adsorbed was determined by fluorescence microscopy. As expected, this study showed that the mass of adsorbed polymer was higher on rough surfaces compared to polished surfaces. The largest relative increase of fluorescence intensity due to PEI as an anchoring layer was observed on silica surfaces, indicating an effect of the substrate on the polyelectrolyte multilayer film properties [27]. Similarly, in our study, we observed that the total amount of polyelectrolytes (PEI-DNA) of six bilayers adsorbed on titanium-coated crystals was ~2 times higher than those on stainless steel-coated crystals [13], confirming the effect of the influence of the substrate surface properties. The most likely influence is a difference in the zeta potential of our surfaces. Still, there could also be minor differences in roughness, leading to the observed difference in adsorbed polymer mass. 

The bacteria-repellant properties of DNA are attributed to the negatively charged phosphate backbone of DNA [13,28]. The Gram-positive staphylococci in this study have a negative surface charge resulting from the teichoic acid grafted on the cell membrane’s peptidoglycan. The negative bacterial surface charge result in a repulsive double-layer interaction with the negatively charged substrate. 

In our earlier study, we showed that DNA-multilayer coatings reduced inorganic and microbial fouling from tap water. The multilayer coatings reduced inorganic fouling from tap water by 90% and impaired biofilm formation by 93% on stainless steel [13]. Here, we demonstrated that CS-DNA polyelectrolyte coatings significantly reduced the biofilm formation of pathogens (*S. aureus* and *S. epidermidis*) on both PMMA and Ti surfaces. *S. aureus* and *S. epidermidis* are the most frequently detected pathogens in implant-associated infections [29]. Similarly, Pingle et al. demonstrated that DNA functionalization significantly reduced *P. aeruginosa* attachment to Si wafers and Si wafers coated with allylamine plasma polymer (AAMpp) [28]. DNA of different molecular weights were immobilized to substrates via both physical adsorption and covalent attachment and were tested against *P. aeruginosa* adhesion at 1 h and 4 h. The authors reported a size-dependent ability to repel *P. aeruginosa*. DNA molecular weights less than 500 bp showed ~70% reduction while molecular weights between 5–15 kbp reduced up to ~82% of the *P. aeruginosa* colonization on both Si and AAMpp surfaces. DNA molecular weights higher than 20 kbp showed the highest *P. aeruginosa* reduction of up to 90% on both surfaces. In another study, Hui et al. demonstrated that nanostructured DNA triangles deposited on SiO_2_ surfaces reduced *B. subtilis* colonization by up to 75% [14]. 

However, the bacteria-repellant property of these and our DNA-functionalized surfaces is likely not a mere function of negative surface charge. It should be noted that the bare substrate surfaces in our study (PMMA and TiO_2_), as well as SiO_2_, are negatively charged after surface cleaning. Furthermore, in a previous study of Gram-negative bacteria, highly negatively charged Niobia surfaces were found to be only bacteria-repellant in the short term [30]. A possible explanation is that the DNA layers are highly hydrated in addition to being negatively charged, thereby reducing protein adsorption and providing an additional entropic penalty to bacteria adsorption.

Chitosan is a cationic polyelectrolyte obtained from different sources, including the shells of crustaceans. It has been extensively investigated and used in pharmaceutical and biomedical applications because of its non-toxic response to tissues and biodegradable properties [31,32]. In addition, chitosan possesses antibacterial activity [31]. The mechanism for the antimicrobial activity of CS is binding to the negatively charged bacterial cell wall, disrupting the cell and altering membrane permeability, followed by inhibition of DNA replication and subsequent bacterial cell death [33,34,35]. Interestingly, in this study, the cationic CS neither promoted bacterial adhesion nor showed antibacterial activity when it was applied as the top layer capping of the four-bilayer CS-DNA film. If a negatively charged surface results in reduced bacteria adsorption due to the repulsive double-layer interactions with the cell surface, then a cationic polymer surface should attract bacteria adsorption [30]. The lack of antibacterial activity of CS could be due to a partial loss of the positive charge on the amino groups at neutral conditions [33,35]. However, the antifouling property of the DNA-containing multilayer coating remained with a CS terminated surface. 

To confirm if the antifouling effect is solely from DNA or also from CS, we investigated biofilm growth of *S. aureus* on PMMA surface coated with three bilayers of PEI-PSS and followed by CS as the top layer. The *S. aureus* biofilm formed on CS terminated PMMA surfaces was similar to on bare PMMA surfaces (Figure S3), indicating that the antifouling property is solely from DNA. This implies a rearrangement of the polyelectrolyte multilayer to present DNA at the interface. However, this hypothesis seems to be at odds with our observation of the linear LbL growth of a fairly rigid film, and the clear change in contact angle when a CS layer terminates the surface. Hence, although the antimicrobial effect correlates entirely with the DNA included in the LbL multilayer, the mechanism of action from these layers remains to be confirmed.

In the cases of orthopedic and dental implants, the establishment of a robust implant-bone interface is essential [6,7]. We observed similar SaOS-2 cell adhesion and spreading on coated compared to uncoated PMMA. These findings suggest that DNA and CS are biocompatible and exhibit no cytotoxic effects on host cells. This adds to the previous conclusion that DNA coatings did not show any cytotoxic effect on the osteoblast-like cells compared to the bare titanium controls [15]. Miyamoto et al. evaluated the in vivo bone responses to multilayered ssDNA/protamine or dsDNA/protamine coating titanium implants in a rat model. Their results showed that higher bone–implant ratios were achieved on DNA-multilayer coatings than on untreated Ti [36]. These previous works combined with our results present a strong case that DNA multilayer coatings can promote osteointegration while simultaneously suppressing the bacteria colonization of an implant surface.

## 5. Conclusions

Stable polyelectrolyte multilayer coatings containing CS-DNA were successfully assembled on titanium and PMMA surfaces using the inexpensive and straightforward layer-by-layer technique. The resulting coatings were well-defined, thin, and had a high elastic modulus compared to previous studies, thanks to the use of a PEI and PSS priming multilayer. We demonstrated that CS-DNA polyelectrolyte multilayer coatings reduced bacterial adhesion and biofilm growth on biomedical implants. The mechanism of suppressing bacterial adhesion was dependent on the DNA component of the coating but not purely relying on electrostatic repulsion. Topping the LbL film with the cationic polymer, chitosan did not hamper the bacteria-repelling properties of DNA. In addition, the DNA-multilayer coatings supported osteoblast-like cell adhesion and spreading. These findings suggest that DNA polyelectrolyte multilayer coatings could be used to improve implant outcomes for, e.g., dental and orthopedic implants.

## Data Availability

The data presented in this study are available within the article or supplementary material.

## Supplementary Materials

The following are available online at https://www.mdpi.com/article/10.3390/ma14164596/s1, Figure S1: Fluorescence microscopic images of SaOS-2 cells after 48 h growth on PMMA, PMMA-LbL (CS-DNA)_4_, and PMMA-LbL(CS-DNA)_4_-CS surfaces. PMMA-LbL(CS-DNA)_4_ are terminated with a DNA top layer, and PMMA-LbL(CS-DNA)_4_-CS are terminated with a CS top layer. SaOS-2 cells were stained with PBS containing DAPI and TRITC-phalloidin. The scale bars denote 25 µm. Figure S2: (A) QCM-D data showing the evolution of the frequency shift, Δf/n, and (B) dissipation ΔD during the film build-up for the overtones n= 3, 5, 7, 9, and 11. The Δf/n decrease observed after each successive layer of polyelectrolytes PEI (yellow dashed lines) and DNA (green) shows a representative example of the film build-up. The Δf/n increase observed after the injection of Milli-Q water (grey dashed lines) indicates the removal of weakly adsorbed excess polymer. The ΔD increased rapidly in tandem with the decrease in Δf/n during layer adsorption. The increase in ΔD was reversed during rinsing, removing the loosely bound polymer. (C) The adsorbed mass of the film calculated according to the Sauerbrey equation as a function of time. Figure S3: Representative fluorescence microscope images of adherent *S. aureus* ATCC 12598 on PMMA and PMMA-LbL(PEI-PSS)_3_-CS surfaces after incubation at 37 °C for 24 h. Bacteria were stained using vitality staining solution (3.34 mM SYTO 9 and 20 mM propidium iodide in PBS) and were incubated for 15 min in the dark at room temperature. The scale bar denotes 25 µm.
