# Supplementary material for: DNA Polyelectrolyte Multilayer Coatings Are Antifouling and Promote Mammalian Cell Adhesion
Source: Materials (Basel). 2021 Aug 16;14(16):4596. doi: 10.3390/ma14164596 (PMC8400194; doi:10.3390/ma14164596)
Supplement: Supplementary file 1 [file materials-14-04596-s001.zip › materials-1316814-supplementary.pdf]

materials

Supplementary Materials

DNA Polyelectrolyte Multilayer Coatings Are Antifouling and Promote Mammalian Cell Adhesion

Omar Abdelaziz Ouni, Guruprakash Subbiahdoss *, Andrea Scheberl and Erik Reimhult

Department of Nanobiotechnology, Institute of Biologically Inspired Materials, University of Natural Resources and Life Sciences (BOKU), 1190, Vienna, Austria; omarabdellazizouni@gmail.com (O.A.O.); andrea.scheberl@boku.ac.at (A.S.); erik.reimhult@boku.ac.at (E.R.)

* Correspondence: guruprakash.subbiahdoss@boku.ac.at (G.S.); Tel.: +43-(1)-47654 (ext. 80205)

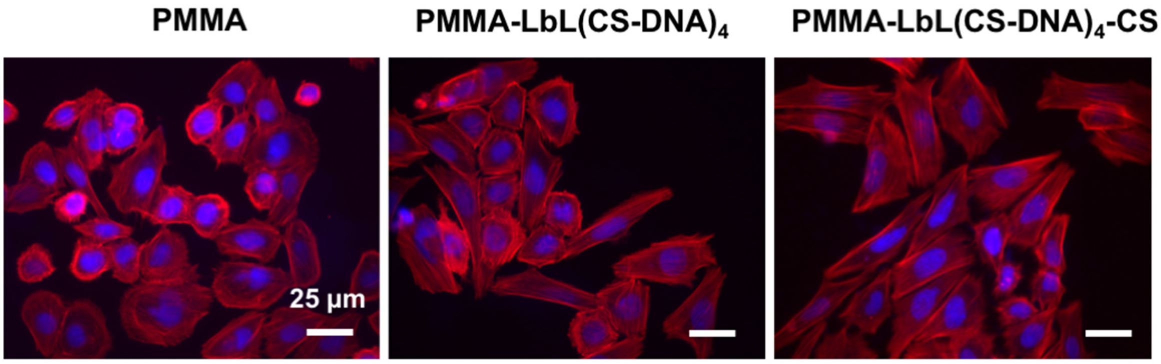

Figure S1. Fluorescence microscopic images of SaOS-2 cells after 48 h growth on PMMA, PMMA-LbL(CS-DNA)$_4$, and PMMA-LbL(CS-DNA)$_4$-CS surfaces. PMMA-LbL(CS-DNA)$_4$ are terminated with a DNA top layer and PMMA-LbL(CS-DNA)$_4$-CS are terminated with a CS top layer. SaOS-2 cells were stained with PBS containing DAPI and TRITC-phalloidin. The scale bars denote 25 µm.

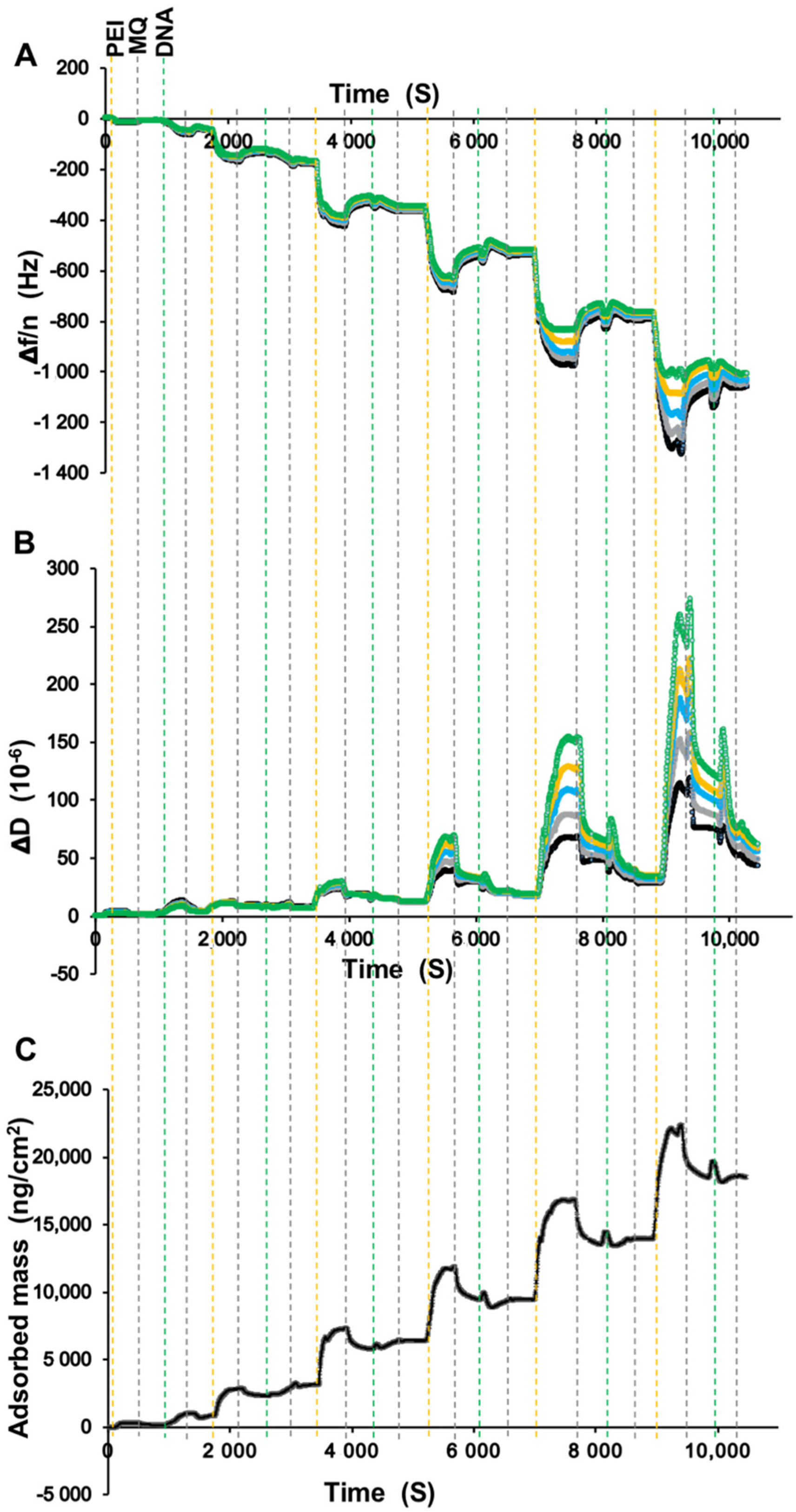

Figure S2. (**A**) QCM-D data showing the evolution of the frequency shift, Δf/n, and (**B**) dissipation ΔD during the film build-up for the overtones n = 3. 5, 7, 9, and 11. The Δf/n decrease observed after each successive layer of polyelectrolytes PEI (yellow dashed lines) and DNA (green) shows a representative example of the film build-up. The Δf/n increase observed after injection of Milli-Q water (grey dashed lines) indicates the removal of weakly adsorbed excess polymer. The ΔD increased rapidly in tandem with the decrease in Δf/n during layer adsorption. The increase in ΔD was reversed during

rinsing, removing loosely bound polymer. C) The adsorbed mass of the film calculated according to the Sauerbrey equation as a function of time.

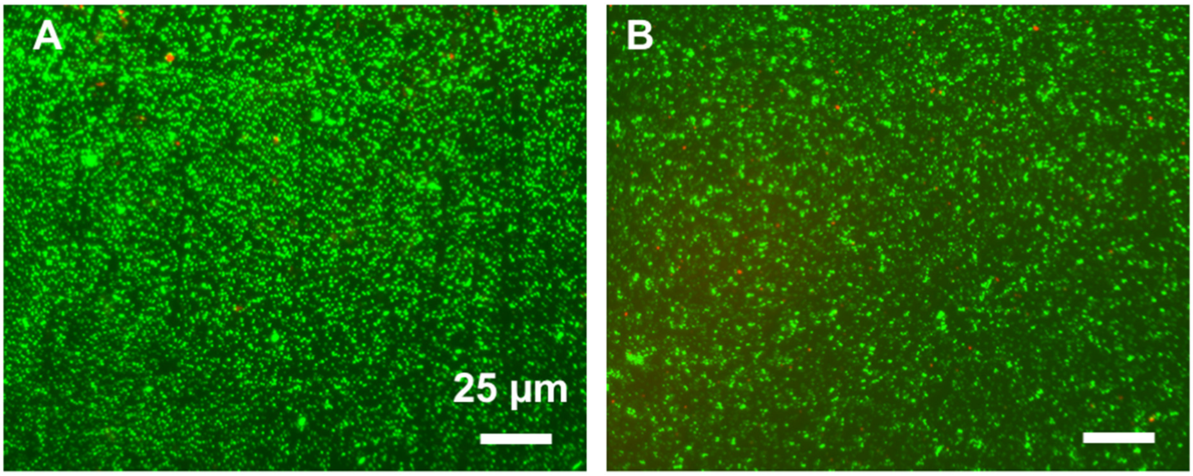

Figure S3. Representative fluorescence microscope images of adherent S. aureus ATCC 12598 on (**A**) PMMA and (**B**) PMMA-LbL(PEI-PSS)3-CS surfaces after incubation at 37 °C for 24 h. Bacteria were stained using vitality staining solution (3.34 mM SYTO 9 and 20 mM propidium iodide in PBS) and incubated for 15 min in the dark at room temperature. The scale bar denotes 25 µm.
